# Neurofibromatosis type 2 misdiagnosed as amblyopia—a case report and literature review

**DOI:** 10.3389/fmed.2025.1556494

**Published:** 2025-08-07

**Authors:** Qian Li, Guiqin Liu

**Affiliations:** ^1^The Second Clinical Medical College of Jinan University, Shenzhen Eye Hospital, Shenzhen, Guangdong, China; ^2^Shenzhen Eye Hospital, Shenzhen, Guangdong, China

**Keywords:** neurofibromatosis type 2, vestibular schwannoma, ophthalmic, OCT, MRI

## Abstract

Neurofibromatosis type 2 (NF2) is characterized by bilateral vestibular schwannomas, and approximately 40–70% of affected children show ophthalmological involvement. Ophthalmological features may be the first sign of NF2; however, the symptoms associated with ocular lesions are diverse. The onset of NF2 is often obscure, resulting in missed or misdiagnosed cases. Herein, we report a case of NF2 in a child who initially presented with poor eyesight. The child had been treated for amblyopia for 7 years and was referred to the eye oncology department due to a lack of improvement in vision. At birth, a soft mass was noted scattered across the abdominal skin and scalp, which gradually increased in size over time. Ophthalmological examination revealed posterior subcapsular opacity in the right eye and an anterior retinal hamartoma in the left eye. Orbital and cranial magnetic resonance imaging (MRI) indicated that the T1 and T2 signals of multiple structures, including the cone, auditory nerve, trigeminal nerve, left oculomotor nerve, paravertebral, and sublingual region, were more uniform after enhancement. A genetic heterozygous mutation was detected, with no family history of the condition. In addition to this case, we collected and summarized 158 publicly reported cases of NF2 with ophthalmological characteristics. Among these cases, the incidence of visual impairment, strabismus, cataract, retinal anomaly, and retinal hamartoma was high, reaching 64, 38, 25, 23, and 16%, respectively. Through analysis and discussion of the clinical and imaging characteristics of NF2 ocular lesions, we aim to improve ophthalmologists’ understanding of this disease, thereby reducing the rate of missed diagnoses.

## 1 Background

Neurofibromatosis type 2 (NF2) is a rare autosomal dominant genetic disease caused by autosomal mutations in chromosome 22q12.2, with an incidence of approximately one in 25,000–60,000, with children accounting for 18% of cases (1, 2). It is generally believed that NF2 develops from benign tumors originating from Schwann cells. This condition often manifests as symptoms developing sequentially in different regions, starting with bilateral or unilateral schwannoma of the cranial nerve, with rare ophthalmic and dermatological involvement (3). Adults with NF2 commonly present with vestibular schwannoma-related hearing loss, tinnitus, and unsteady gait; however, the initial symptoms in children are atypical, with ophthalmic and skin tumors being plausible primary manifestations (4, 5). However, due to the obscure onset of this disease, ophthalmic symptoms are sometimes missed. This article reports a case of NF2 in a child who initially presented with vision loss and abdominal wall swelling, and was misdiagnosed with amblyopia, for which treatment continued for 7 years. In addition, we provide a summary of the relevant literature, analyzing and summarizing the clinical and imaging characteristics of NF2 intraocular lesions, as well as the treatment progress of this condition.

## 2 Case introduction

A 10-year-old girl presented to Shenzhen Eye Hospital in June 2023 due to “blurred vision for more than 7 years.” When the child was 3 years old, she was found to exhibit bilateral visual disturbance by her parents. She was subsequently diagnosed with amblyopia in the local hospital, for which she underwent treatment for 7 years. However, her eyesight did not improve, so she was referred to the ocular oncology department for further examination.

**Figure 1:**
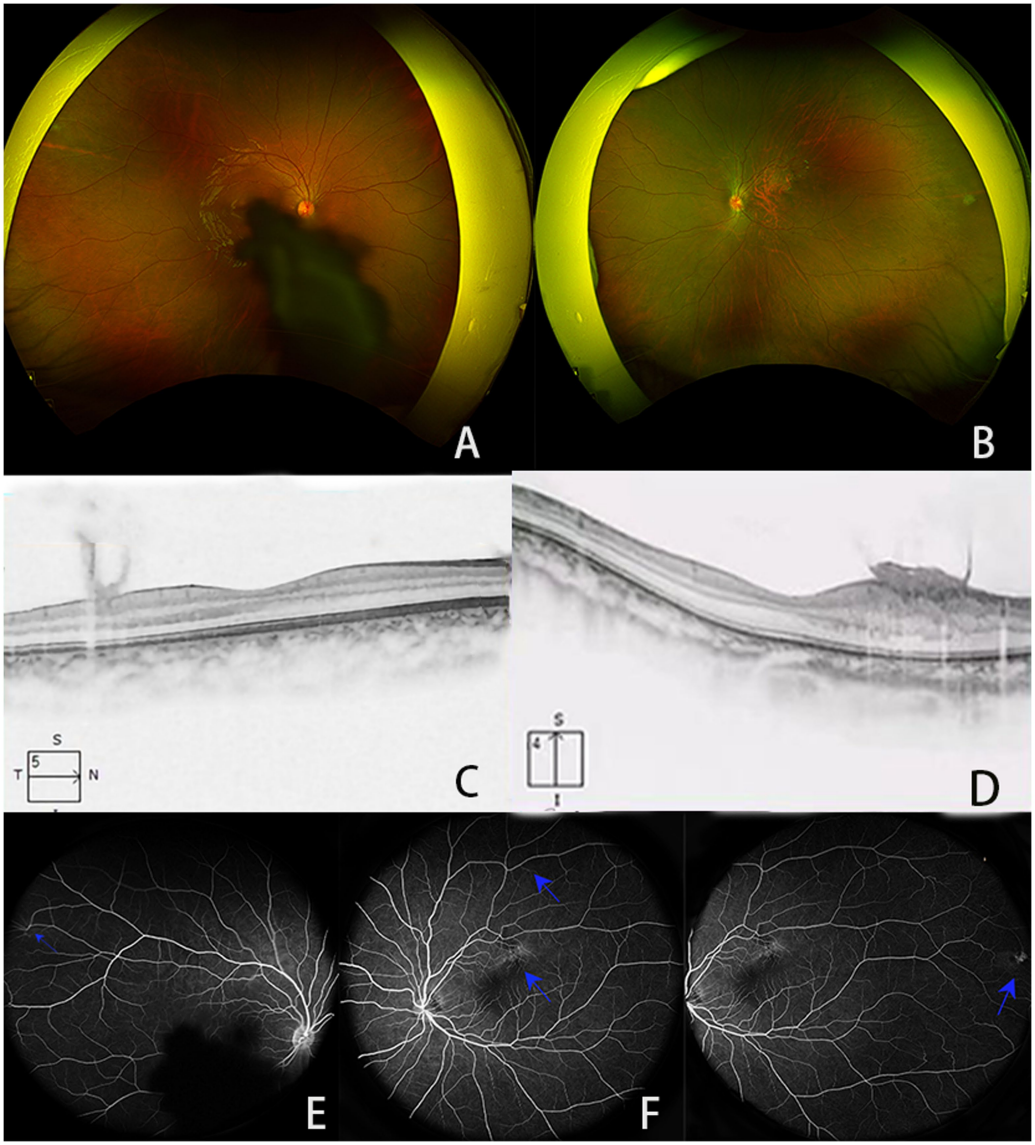
**(A,B)** Results of scanning laser fundusography; **(C,D)** images of the macular area, showing **(C)** medium- and low-signal membranes in front of the concave temporal nerve epithelium in the center of the macula of the right eye and **(D)** the nerve epithelial layer above the concave temporal in the center of the macula of the left eye showed thickening, the boundaries of each layer were unclear, and the surface signal was rough, which could be seen as a flame-like membrane attachment; **(E–G)** FFA images showing **(E)** the refractive interstitial turbidity of the posterior pole of the right eye is obscured with fluorescence, and localized vascular tortuosity is located in the temporal side (blue arrow), **(F)** the superior temporal arteriovenous branch above the macula of the left eye is roundabout, with mild fluorescence accumulation with the angiography process (blue arrow), showing a clear boundary and intense fluorescence. The local fluorescence of the retina above the lesion is low (blue arrow), **(G)** the retinal blood around the temporal side of the left eye is bent and expanded, and the temporal side is accompanied by small pieces of transparent masking fluorescence; optic disc fluorescence enhancement (blue arrow).

Physical examination revealed a 1.3 cm × 1.3 cm soft, flesh-colored tumor on the right scalp, along with three finger-sized, soft, flesh-colored tumors on the abdomen. Ocular examination showed visual acuities of 0.5 and 0.3 in the right and left eyes, respectively. Slit lamp examination revealed no obvious abnormalities in the conjunctiva, cornea, or anterior chamber; however, the right lens showed turbidity under the posterior capsule. Fundoscopic examination indicated that the boundary of the disc of the right eye was clear, the retina in the macular area was blocked by a membrane (Figures 1A,B). No obvious abnormalities were observed at the bottom of the left eye. B-mode ultrasonography revealed an oval medium-to-low echo shadow on the temporal side of the optic nerve of the left eyeball, with well-defined boundaries. Optical coherence tomography (OCT) revealed no obvious abnormalities in the concave shape of the macula center. A medium-to-low signal membrane was observed in front of the concave temporal nerve epithelium in the center of the right eye. The nerve epithelium above the concave temporal in the center of the left eye was thickened, the boundaries of each layer were unclear, and the surface signal was rough, which was observed as a flame-like membrane attachment, with anterior projection into the vitreous and hazy borders (Figures 1C,D). Fluorescein angiography (FFA) revealed interstitial turbidity of the posterior polar refraction of the right eye, resulting in concealed fluorescence. Additionally, localized blood vessels on the temporal side appeared detoured. Specifically, the upper temporal arteriovenous branch in the upper macula of the left eye was deformed, showing accumulation over time, with unclear boundaries and intense fluorescence. Furthermore, the local fluorescence in the upper retina above the lesion was reduced. Peripheral retinal blood vessels appeared tortuous, and the temporal side was accompanied by a small area of blocked fluorescence. Finally, disk fluorescence was enhanced (Figures 1E–G).

**Figure 2:**
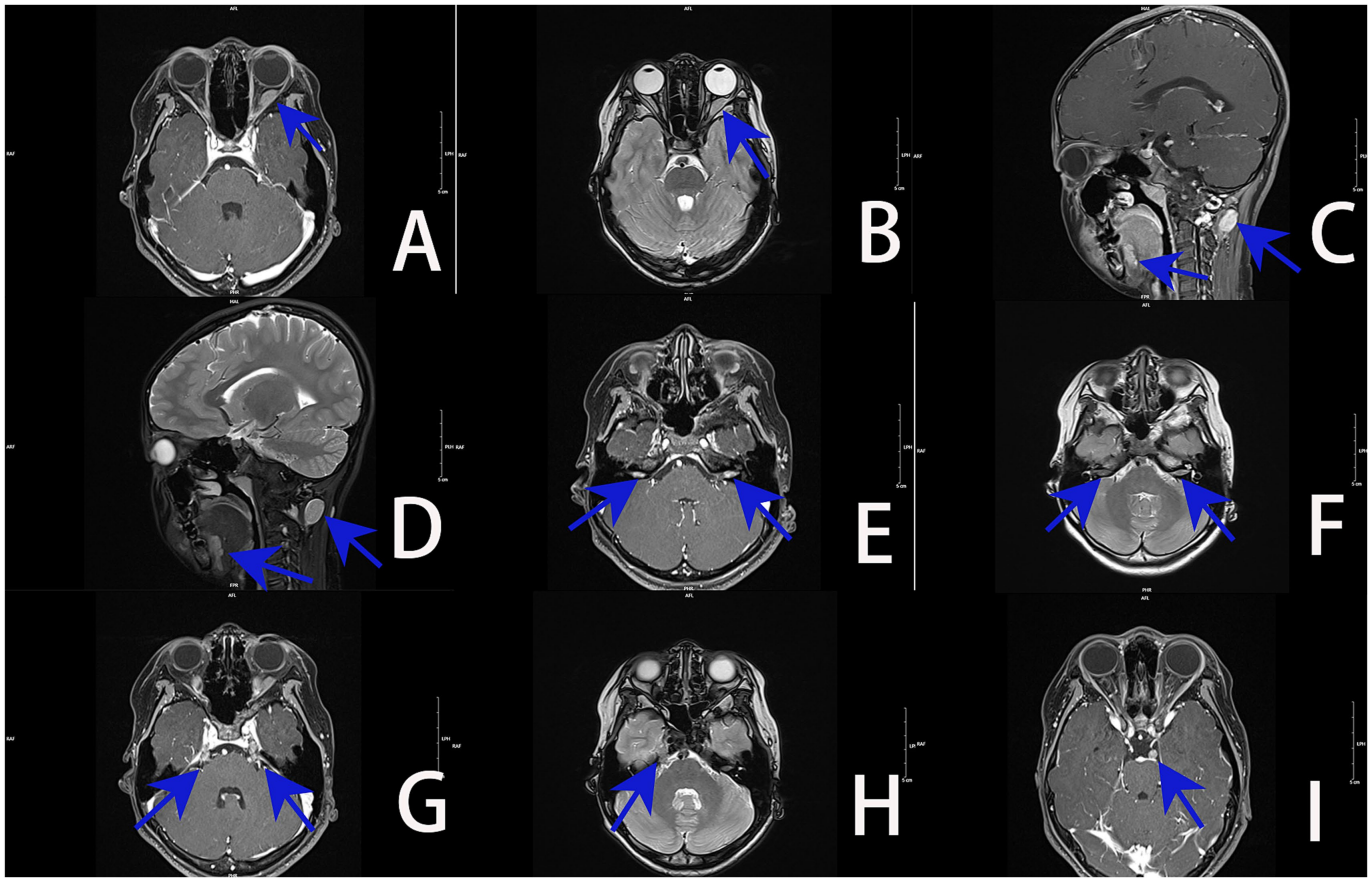
MRI images of the orbit and brain (blue arrow denotes the lesion): **(A)** irregularly shaped lesion with homogeneous T1 enhancement in the left intraconal space adjacent to the lateral rectus muscle. **(B)** Left intraconal tumor demonstrates mildly prolonged T2 signal, with posterior extension into the optic canal and mild thickening of the optic nerve. **(C)** Ovoid T1-enhancing lesion at the posterior left neck, with irregular abnormal enhancement at the anterior inferior tongue. **(D)** Posterior left neck shows mildly prolonged T2 signal; anterior inferior tongue demonstrates heterogeneous T2 hyperintensity. **(E)** Bilateral cerebellopontine angles exhibit irregular lesions with relatively homogeneous T1 enhancement; no significant expansion of the internal auditory canals. **(F)** Bilateral cerebellopontine angles show irregular foci of T2 hyperintensity. **(G)** Bilateral trigeminal nerve courses demonstrate heterogeneous T1 enhancement. **(H)** Right trigeminal nerve pathway shows irregular foci of T2 hyperintensity. **(I)** Ovoid homogeneous T1-enhancing lesion along the left parasellar oculomotor nerve course (arrow indicates).

Magnetic resonance imaging (MRI) of the orbit and brain revealed an irregular space-occupying lesion in the cone of the left orbital muscle close to the external rectus muscle. This tumor showed equal signals, with slightly longer T1 and T2 signals. Following the enhancement, an obvious even strengthening was observed, with a range of approximately 28 × 5.8 mm. Its back pole extended to the optic nerve tube, while the optic nerve was slightly thicker. The back of the left neck showed no increase with T1 but showed a slightly longer T2 signal, with uneven strengthening, in a region approximately 19 mm × 15 mm in size. The signal was uneven in the front of the lower tongue, with irregular and slightly longer T2 signals. Irregular T1 was uniformly enhanced in the horn region of the bilateral cerebellar, and there was no significant expansion of the auditory canal. Uneven enhancement was observed in the walking area of the bilateral trigeminal nerves show T1 heterogeneous enhancement along their course. The left sellar oculomotor nerve course presents an elliptical T1 homogeneous enhancement. Imaging diagnosis of these space-occupying lesions: Schwannoma (Figure 2).

**Figure 3:**
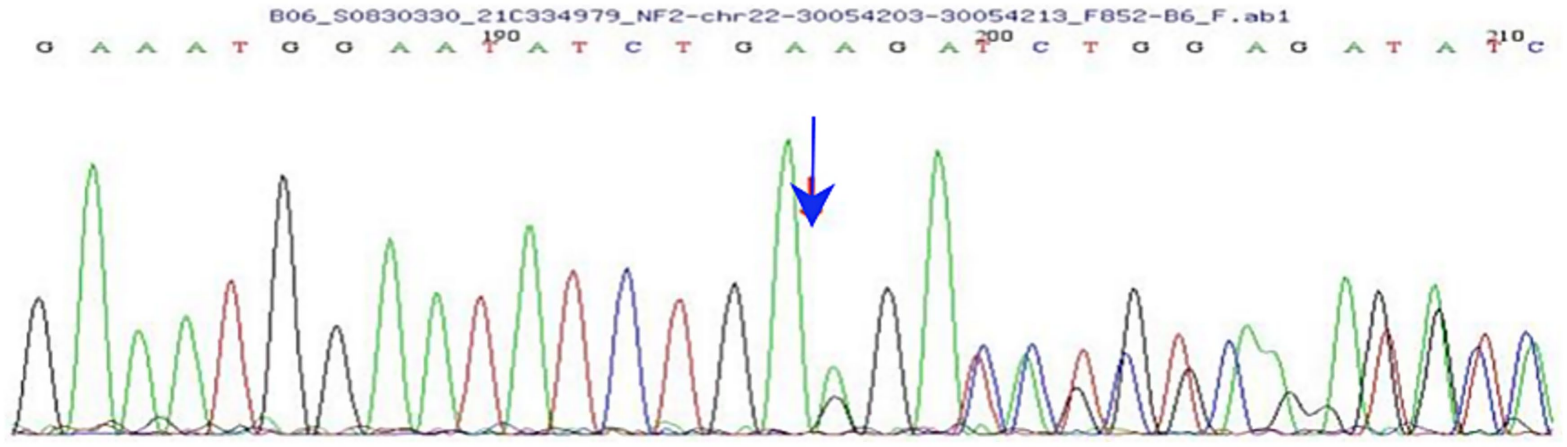
Results of whole-exon second-generation sequencing show a heterozygous mutation on chromosome 22 (arrow indicates the mutation).

Further questioning revealed that the child had experienced chronic swelling in the lower abdomen since early childhood. She visited Shenzhen Children’s Hospital in September 2021, where a schwannoma was suspected. Whole-exon second-generation sequencing of the genomes of the proband (Figure 3), her parents, and her sister showed no pathogenic variants in the parents or sister, indicating a normal genotype. However, further analysis revealed a heterozygous mutation on the child’s 22nd chromosome (c.626_635del AGATA GCTCA), leading to a frameshift mutation in amino acids. This was identified as a spontaneous mutation. No pathogenic variants at this site have been previously reported in the literature or relevant databases. In June 2023, a tumor biopsy of the lower abdomen was performed, and the pathological diagnosis confirmed NF2. The child had a homozygous mutation in the NF2 gene, which caused a frameshift and was identified as a spontaneous mutation. Neither the literature nor relevant databases have reported any correlation for this site. The case data of this patient have undergone ethical review by the Shenzhen Eye Hospital Medical Ethics Committee of Shenzhen Eye Hospital (Approval number: 2024KYPJ141).

## 3 Discussion

NF2 is a rare multiple tumor syndrome with no gender-based disparities, and its incidence rate in newborns is approximately 1 in 25,000 (6). This condition was first described by Wishart (7). The NF2 gene contains 17 coding exons that encode a protein called Merlin, which is distributed in Schwann cells, meningeal cells, lenses, and other nerve tissues (8). Merlin primarily mediates interactions between actin filaments, intracellular signal carriers, and membrane proteins. It contributes to the stability of the membrane by regulating cell growth and differentiation and exerts a tumor-suppressive function. Inactivation of Merlin can lead to uncontrolled mitotic signaling and tumor growth (8, 9). Currently, there are two theories regarding the pathogenesis of NF2: (1) the two-hit model: this model postulated that the occurrence of tumors originates from allele mutations, i.e., mutations in both alleles, or a mutation in one allele and loss of the other allele, resulting in a decrease in Merlin protein expression or structural abnormalities; and (2) epigenetic mechanisms: this hypothesis posits that the NF2 gene is epigenetically modified through methylation, miRNA expression disorders, and other epigenetic modifications, resulting in a decrease in Merlin protein expression, thereby preventing it from playing a normal role in tumor inhibition (10). Because there are hundreds of mutations in the inhibitory gene of NF2 tumors, it can manifest as neurosheath tumors of the central and peripheral nervous systems, multiple meningiomas, ventricular meningiomas, and other related forms. Individuals with NF2 generally present with bilateral vestibular schwannomas. In addition, lenticular opacities, retinal hamartoma, epiretinal membranes, and other characteristics may appear in the eyes, while coffee spots and fibromas may appear on the skin (11). The Manchester diagnostic criteria divide NF2 into three phenotypes, based on the clinical manifestations: (1) Gardner NF2: this type presents with mild clinical symptoms and late onset and generally only manifests as bilateral vestibular neurinoma; (2) Wishart NF2: this form can present in childhood, manifests as a bilateral vestibular schwannoma and multiple central nervous system tumors, as well as skin and eye symptoms; (3) segmental: this subtype can appear as a homolateral vestibular schwannoma with meningioma, or multiple Schwann tumors limited to part of the peripheral nervous system (12).

Peyre et al. (13) reported that approximately 90–95% of NF2 cases are accompanied by bilateral vestibular schwannomas, Adults typically present with bilateral vestibular schwannomas, often accompanied by multiple meningiomas or neurogenic tumors, while at least two-thirds of patients ultimately develop spinal tumors (14). The typical sign of an NF2 vestibular schwannoma in magnetic resonance imaging (MRI) is an occupied lesion of the cerebellopontine angle, centered on the inner auditory canal, but potentially extending and enlarging beyond it. These lesions appear as low-to-moderately intense on T1WI, with slightly increased intensity on T2WI. Enhancement on scanning is uneven, and some tumors exhibit cystic changes and necrotic areas, characterized by long T1 and T2 signals; however, the cystic deformations are not strengthened (9, 15, 16). Meningiomas and ependymomas are also important manifestations of NF2. Meningiomas generally present as marginally clear masses, which can occur in any region of the brain, but are most commonly found in the prefrontal area of the frontal lobe, the parietal lobe, the temporal area, and along the falx cerebri. Some meningiomas show meningeal tail signs, manifesting as equal intensity on T1 and T2 MRI, with significant enhancement. Ependymomas most commonly involve the posterior cranial fossa and cervical marrow region of the spine, which can cause different degrees of clinical symptoms, including limb weakness and numbness (9, 17).

In children, NF2 often presents with ophthalmic involvement. Filizoglu et al. (6) proposed that the disease course of children is distinct from that of adults and that ophthalmic symptoms typically occur before neurological symptoms and hearing loss. In this study, we searched using the key terms “Neurofibromatosis Type 2,” “Type 2 Neurofibromatosis,” “acoustic neurofibromatoses,” “NF2,” and “neurofibromatosis” to identify full-text articles in Chinese and English detailing eye performance records of NF2 published from 1995 to 2022. Two ophthalmologists evaluated the retrieved articles. Based on the specific content and description of ocular conditions in the literature, a total of 19 articles involving 158 patients with NF2-related ocular lesions were identified (Table 1). Reported ocular symptoms include ptosis, nystagmus, corneal damage, impaired vision, strabismus, cataracts, retinal diseases, and intraorbital space-occupying lesions. Among them, the incidences of visual impairment, strabismus, cataract, epiretinal membrane (ERM), and retinal malformation were relatively high, reaching 64, 38, 25, 23, and 16%, respectively.

**Table 1:** Summary of previously published case reports of NF2 abnormal ophthalmological symptoms (*n* = 158 cases).

Author	Publication period	Age	Gender	Number of cases	Primary ophthalmological symptoms
Kunikata et al. (18)	2022	10 years old	F	1	Vision of the right eye is impaired; the right eye had a thickened epiretinal membrane; the foveal contour was lost.
Wishart et al. (7)	2021	7 years old	F	1	Severe left microphthalmia; glaucoma; total retinal detachment in the left eye.
Zarei et al. (19)	2020	22 years old	F	1	Bilateral posterior subcapsular and cortical cataract; optic disk swelling; hyperpigmented macular lesions; epiretinal membrane; macular temporal dragging.
Luowen et al. (20)	2020	10 years old	M	1	Swelling of the upper eyelid of the right eye; round swelling at the root of the nose; presence of an intraorbital space-occupying lesion of the right eyeball.
Giovinazzo et al. (21)	2019	8 years old	F	1	Intermittent exotropia; eccentric preretinal lesions in the maculas; and loss of the foveal contour.
Waisberg et al. (22)	2019	14–50 years old	–	8	Cataract, strabismus; chronic retinal detachment; retinal hamartoma; epiretinal membrane; and choroidal nodules.
Anand et al. (5)	2018	0–16 years old	–	32	Visual impairment; squint; nystagmus, ptosis; unilateral optic atrophy; optic nerve morning glory syndrome; hamartoma; epiretinal membrane; and cataract.
Kang et al. (23)	2018	20 years old	M	1	Vision impairment; cataract; unclear optic nipple boundaries; and retinal edema.
Waisberg et al. (24)	2016	14–50 years old	–	9	Cataract; epiretinal membrane; chronic retinal detachment; retinal malformation; optic disc edema; and optic nerve atrophy.
Mahroo et al. (25)	2016	18 years old	F	1	Cilioretinal artery territory infarction with papilledema
Kang et al. (35)	2013	5 years old	F	1	Cataract; combined hamartoma of the retina and retinal pigment epithelium; epiretinal membrane; retinal malformation; and posterior vitreous detachment with retinal folds.
Han et al. (27)	2012	2 years old	F	1	Esotropia and amblyopia in the left eye; epiretinal membrane of the left fundus retina; and macular decoloration lesions.
Feucht et al. (28)	2008	–	–	73	Strabismus; amblyopia; nystagmus; anisometropia; refractive error; and cataracts.
Kim et al. (29)	2005	3-month-old	M	1	nystagmus; variable esotropia; constant nystagmus; posterior subcapsular cataracts; and epiretinal membrane with significant vascular distortion.
Lihua et al. (30)	2005	14–55 years old	–	4	Decreased vision; eyeball protrusion with pain; strabismus; pale nipple color; and intraorbital space-occupying lesions.
Berney et al. (31)	2004	82 years old	F	1	Protrusion of the left eyeball; exposed keratitis; the temporal part of the fundus optic disc is slightly pale; and multiple space-occupying lesions inside and outside the orbit.
Ragge et al. (32)	1997	15–59 years old	–	5	Eyeball indentation; upper eyelid ptosis; congenital internal strabismus; posterior cystic cataract; retinal dislocation; epiretinal membrane with macular displacement; optic nerve atrophy; and intraorbital-occupying lesions.
Rettele et al. (33)	1996	21 years old	M	1	Loss of vision, right pupil showing slowed reaction time, the right retina disclosed a nonexcavated, yellowish, triangular, and retinochoroidal defect incorporating the optic disk superiorly and extending to just beyond the temporal vascular arcade inferiorly
Meyers et al. (34)	1995	–	–	15	Epiretinal membrane in the macular or paramacular area; combined pigment epithelial and retinal hamartoma in the macula; epiretinal membrane; and central. Posterior cortical, subcapsular, or peripheral cortical lens opacities; and retinal upper temporal to macular depigmentation.

F, Female; M, Male.

Previous studies have indicated that more than 40–70% of children with NF2 have cataracts, retinopathy, or both. Among these children, cataracts are the primary clinical manifestations of NF2, usually hidden behind posterior subcapsular cataracts (26, 35), while retinal diseases include flame-shaped ERM, retinal tufts, and retinal micro-hamartomas (22). OCT studies have generally shown that NF2 retinal hamartoma mainly affects the inner layer of the retina, while ERMs in NF2 manifest as an irregular and hyperreflective layer of the inner limiting membrane (36). In addition, NF2 can also cause optic disc swelling, macular lesions, macular dragging, focal choroidal excavation, optic nerve tumors, and other optic nerve-related abnormalities (19, 21). Waisberg et al. (24) previously suggested that flame-shaped ERM is a specific finding of NF2 and could be considered an important diagnostic sign. Studies performing clinical observations have found that the ocular features of NF2 in children may appear earlier and that patients with vision loss in childhood have an increased risk of multiple tumors in the central nervous system, and their related incidence and mortality rates are usually higher than those of adults with NF2 (11, 37). The latest international consensus recommendation in 2022 revised the diagnostic criteria for NF2 to state that diagnosis can be performed when the patient meets any of the following conditions: (1) presence of bilateral vestibular schwannomas; (2) presence of at least two anatomically distinct NF2-related tumors (schwannoma, meningioma, and ependymoma); (3) meeting either two major or one major and two minor criteria, defined as follows: major criteria: unilateral vestibular schwannomas, two or more meningiomas, a first-degree relative—other than a sibling—with NF2-related schwannomatosis and minor criteria: ependymoma, meningioma, schwannoma, juvenile subcapsular or cortical cataract, retinal dislocation, epiretinal membrane in patients under 40 years of age, or a single meningioma (11).

NF2 is a rare disease, and 50% of NF2 patients have no relevant familial genetic history. The child in the present case showed visual impairment, with no abnormalities in eye appearance. The missed diagnosis rate of NF2 is high when detailed examinations are not performed. Previous studies have calculated that the average delayed diagnosis times in children with NF2 are 6.7, 7, and 11 years (19, 21, 38).

Bilateral visual impairment was identified when the patient was 3 years old, at which point amblyopia treatment started, and an abdominal mass was found at the same time, without specific treatment. Pathological biopsy and gene test of the abdominal mass were performed at the children’s hospital at the age of 8 years, and NF2 was confirmed. The patient was referred to our hospital when she was 10 years old due to poor binocular visual correction. The patient was referred to the Department of Orbital Disease and Ocular Tumor. The patient was diagnosed with amblyopia for many years, and one of the reasons was that the pediatric disease specialist ignored the binocular visual abnormality, which delayed the diagnosis. In contrast, although studies show that the prevalence of amblyopia in children is 2–4%, making it the most common cause of one-eyed vision impairment in children and adolescents, it is very hasty for doctors to diagnose amblyopia without a detailed eye examination. Additionally, awareness of NF2 remains very low (39). Amblyopia doctors do not know NF2, which eventually leads to misdiagnosis of children.

Amblyopia is defined as an abnormality during the visual development period, due to strabismus, uncorrected refractive aberration, or high refractive error or shape deprivation, resulting in a best-corrected visual acuity below the normal level or a difference between the two eyes of more than two lines (40, 41). Amblyopia can only be diagnosed after various causes of vision loss are excluded. Children with normal symptoms but poor effect after amblyopia training require further examination. Several studies have reported that children with keratoconus (42), congenital cataracts, congenital macular abnormalities, foveal retinoschisis, and brain-derived vision impairment (42–44) have been misdiagnosed with amblyopia.

In addition to the above diseases, diseases that have a normal appearance but may affect children’s vision include congenital refractive errors, congenital keratoconus, congenital glaucoma, retinoblastoma, Coat’s disease, retinopathy of prematurity, persistent hyperplastic primary vitreous, optic neuritis, Leber’s hereditary optic neuropathy, congenital retinopathy, optic nerve dysplasia, compressive optic neuropathy caused by optic nerve, and orbital and intracranial space occupation. The essence of amblyopia is the absence of organic lesions, and the best-corrected visual acuity is lower than the standard for the same age. If there is structural normality, other disease diagnoses should be considered. The abovementioned organic diseases can be differentiated through fundus examination, refraction, corneal topography, intraocular pressure measurement, electrophysiological examination, and MRI. The main factors causing vision loss in the present child were a cataract in the right eye, a retinal hamartoma in the left eye, and an epiretinal membrane in the binocular region. In this case, the right epiretinal membrane was far from the center of the macula, and the cataract only covered part of the axis of sight. At the same time, the epiretinal membrane in the left eye was closer to the center of the macula, and the interlayer structure of the retina was more harmful to the vision, meaning that the naked eye vision of the patient’s left eye was worse than that of the right eye. The schwannoma adjacent to the rectus muscle, both inside and outside the left orbital cone, is situated at a distance from the optic nerve and therefore has little impact on the optic function.

The U. S. Preventive Services Task Force recommends screening children aged 3–5 for amblyopia or risk factors (45). Clinicians should remain vigilant for reduced visual acuity in the absence of strabismus or fundus abnormalities, particularly when systemic manifestations are present. Indicators such as cutaneous tumors, growth retardation, neurological symptoms, and other signs may suggest central nervous system lesions or genetic disorders, warranting MRI examinations. Meyers et al. (34) believed that children or young patients with epiretinal membranes, combined pigment epithelial retinal hamartoma, and lens opacities that are not the result of other ocular disorders should have a neurologic evaluation and a careful evaluation of family history for NF2. Therefore, brainstem auditory evoked potential (BAEP) plays a pivotal role in auditory assessment. A multidimensional diagnostic approach is essential for the accurate diagnosis of complex ocular diseases. Relying solely on routine ophthalmic examinations may lead to missed diagnoses.

Drug therapy represents a critical intervention for pediatric patients with NF2. As an anti-vascular endothelial growth factor (VEGF) monoclonal antibody, bevacizumab exerts its efficacy by inhibiting VEGF-mediated tumor angiogenesis, thereby alleviating compressive symptoms such as hearing loss. Notwithstanding, this agent is associated with notable adverse events, including hypertension, and tumor recurrence is frequent upon treatment discontinuation. Concerns also exist regarding its potential impact on pediatric growth and development. Multikinase inhibitors remain in the exploratory phase of clinical evaluation. Mammalian target of rapamycin (mTOR) inhibitors, typified by sirolimus, demonstrate efficacy in tumor progression control but are not devoid of adverse reactions (46, 47). It is important to note that bevacizumab has not yet received official approval from regulatory authorities, such as the U. S. Food and Drug Administration (FDA), for NF2 indications. The child’s current binocular corrected visual acuity remains 0.6. As the orbital tumor has not yet compressed the optic nerve, the guardians have opted for conservative management. Surgical intervention may be considered in the presence of complications such as cataract, vitreous membrane prolapse, or intracranial tumors.

Children with NF2 have lifelong, unpredictable tumor growth potential. Giovanizzio et al. (21) reported the case of a child diagnosed with strabismus, in whom the central concave contour of the binocular macular retina had been completely lost. In this case, the symptoms of vision loss appeared earlier, with a risk of further vision decline if the retinal disease continued to develop. Although the patient displayed no obvious signs of vestibular neurinoma, such as hearing loss, headache, vomiting, and ataxia, MRI revealed bilateral auditory neuroma and multiple peripheral and central nervous system tumors, which is consistent with severe (Wishart type) NF2, with a poor prognosis. This type is characterized by early onset and rapid progression of multi-system tumors, and the prognosis is significantly worse than that of the classic type.

Children with NF2 must undergo orbital MRI and hearing tests at least once a year to track the progress of the disease (9), and if vision is significantly reduced, surgical intervention may be considered to remove the epiretinal membrane (17). At present, the child’s binocular corrective vision acuity is 0.6. After a year of follow-up, there were no symptoms such as hearing loss or ataxia. In May 2025, the child re-examined the MRI of the craniocerebral and neck and improved the MRI of the lumbar and thoracic vertebrae. There was no noticeable change in craniocerebral and eye tumors. Multiple nodules were discovered in the child’s thoracic and lumbar vertebrae, accompanied by sensory disturbances and mild muscle atrophy in the right lower leg. Neurosurgeons recommend a partial resection of the lumbar nodules to alleviate the atrophy in the right leg. Postoperatively, close follow-up is necessary, with regular MRI scans of the thoracolumbar spine, orbits, and cranium to monitor the size and progression of acoustic neuromas and other intracranial/intraspinal neuromas. Additionally, one should observe for new symptoms, such as hearing loss, tinnitus, and facial numbness, as well as any further neurological impairment.

## 4 Conclusion

NF2 is a genetically caused disease that manifests as multiple tumors in both the central and peripheral nervous systems. Detailed screening of ocular lesions and whole-spine imaging are important for diagnosis and prognosis (26). The limitation of this article is that the child did not undergo imaging of the thoracic and lumbar spine when he first came to our department. Although thoracic and lumbar spine MRI was performed during the follow-up period, no comparison or further analysis of the genetic findings was conducted. In addition, as this is a case report, the follow-up period is relatively short. In the future, we plan to conduct long-term follow-ups to improve our understanding of the progression of ocular and systemic conditions of NF2 and to jointly develop future diagnostic and treatment plans with neurologists.

## Data Availability

The original contributions presented in the study are included in the article, further inquiries can be directed to the corresponding author.
